# Report of International American Conference

**Published:** 1890-11

**Authors:** 


REPORT AND RECOMMENDATIONS OF INTERNATIONAL AMERICAN CONFERENCE UPON POSTAL AND CABLE COMMUNICATION WITH CENTRAL AND SOUTH AMERICA.

This pamphlet is a recommendation for a government subsidy of a telegraphic cable in the Pacific Ocean between San Francisco and Valparaiso in Chili, and for promoting postal communication.

